# Binocular stereo-navigation for three-dimensional thoracoscopic lung resection

**DOI:** 10.1186/s12893-015-0044-y

**Published:** 2015-05-08

**Authors:** Masato Kanzaki, Tamami Isaka, Takuma Kikkawa, Kei Sakamoto, Takehito Yoshiya, Shota Mitsuboshi, Kunihiro Oyama, Masahide Murasugi, Takamasa Onuki

**Affiliations:** Department of Surgery I, Tokyo Women’s Medical University, 8-1 Kawada-cho, Shinjuku-ku, Tokyo, 162-8666 Japan

**Keywords:** Computed tomography, Video-assisted thoracic surgery, Thoracoscopic sublobar resection, Three-dimensional imaging, Binocular stereo-navigation

## Abstract

**Background:**

This study investigated the efficacy of binocular stereo-navigation during three-dimensional (3-D) thoracoscopic sublobar resection (TSLR).

**Methods:**

From July 2001, the authors’ department began to use a virtual 3-D pulmonary model on a personal computer (PC) for preoperative simulation before thoracoscopic lung resection and for intraoperative navigation during operation. From 120 of 1-mm thin-sliced high-resolution computed tomography (HRCT)-scan images of tumor and hilum, homemade software CTTRY allowed sugeons to mark pulmonary arteries, veins, bronchi, and tumor on the HRCT images manually. The location and thickness of pulmonary vessels and bronchi were rendered as diverse size cylinders. With the resulting numerical data, a 3-D image was reconstructed by Metasequoia shareware. Subsequently, the data of reconstructed 3-D images were converted to Autodesk data, which appeared on a stereoscopic-vision display. Surgeons wearing 3-D polarized glasses performed 3-D TSLR.

**Results:**

The patients consisted of 5 men and 5 women, ranging in age from 65 to 84 years. The clinical diagnoses were a primary lung cancer in 6 cases and a solitary metastatic lung tumor in 4 cases. Eight single segmentectomies, one bi-segmentectomy, and one bi-subsegmentectomy were performed. Hilar lymphadenectomy with mediastinal lymph node sampling has been performed in 6 primary lung cancers, but four patients with metastatic lung tumors were performed without lymphadenectomy. The operation time and estimated blood loss ranged from 125 to 333 min and from 5 to 187 g, respectively. There were no intraoperative complications and no conversion to open thoracotomy and lobectomy. Postoperative courses of eight patients were uneventful, and another two patients had a prolonged lung air leak. The drainage duration and hospital stay ranged from 2 to 13 days and from 8 to 19 days, respectively. The tumor histology of primary lung cancer showed 5 adenocarcinoma and 1 squamous cell carcinoma. All primary lung cancers were at stage IA. The organs having metastatic pulmonary tumors were kidney, bladder, breast, and rectum. No patients had macroscopically positive surgical margins.

**Conclusions:**

Binocular stereo-navigation was able to identify the bronchovascular structures accurately and suitable to perform TSLR with a sufficient margin for small pulmonary tumors.

## Background

As computed tomography (CT) technology has advanced, the early detection of small and peripheral pulmonary nodules including early stage lung cancer have markedly increased. For these pulmonary nodules, lung sublobar resection becomes a favorable option and can yield promising outcomes over those of lobectomy [1–5]. Since the cases of Japanese lung cancers more than half are operated by video-assisted thoracic surgery (VATS), thoracoscopic sublobar resection (TSLR) is often performed for treating early stage lung cancer [6]. Because of many variations found in the structures of the pulmonary vessel branches and segmental bronchi, TSLR requires surgeons to have more experience and skill for performing the surgical procedure safely. Since the three-dimensional (3-D) images of lung structures recently can be prepared easily [7–10], based on patient’s personal 3-D imaging, presurgical planning is performed, and intraoperative navigation is available during thoracoscopic lung resection [11]. Previously, our department has reported thoracoscopic lung resections guided by patients’ personal virtual 3-D pulmonary models using homemade software [12–14]. In this report, the authors investigated the efficacy of binocular stereo-navigation during 3-D TSLR.

## Methods

**Fig. 1:**
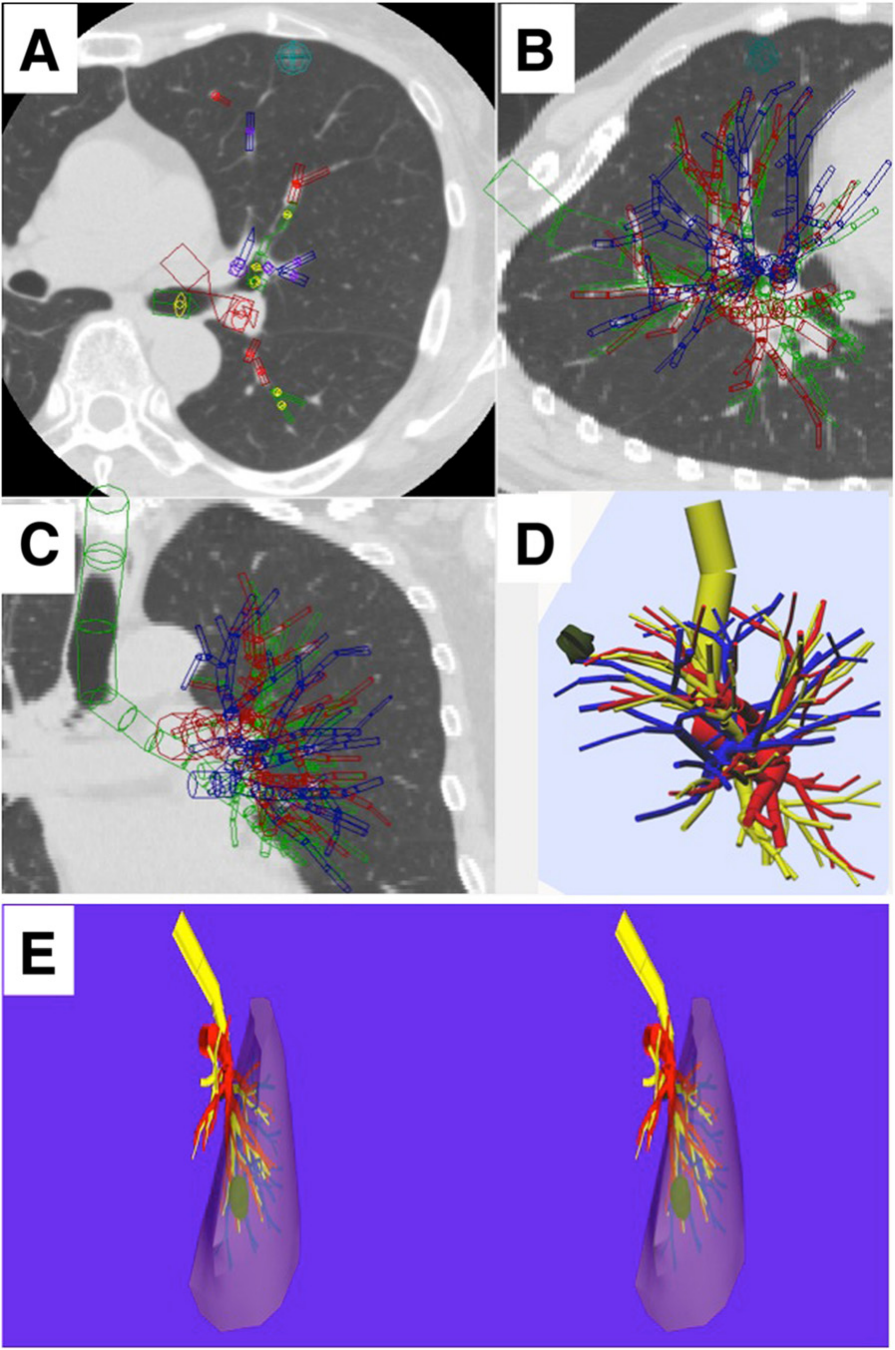
The creation of patient-actual virtual 3-D pulmonary model. (**a-c**) After the communications-in-medicine (DICOM) format images, which were obtained from 120 of 1-mm thin-sliced high-resolution computed tomography (HRCT)-scan images of tumor and hilum, were uploaded to a personal computer (PC), homemade software “CTTRY” (Tokyo Women’s Medical University, Tokyo, Japan) allowed surgeons to mark pulmonary arteries, veins, bronchi, and tumor on the HRCT image manually and attempt to reconstruct an anatomical model with the help of anatomically correct images. (**d**) The locations and thicknesses of the pulmonary vessels and bronchi were rendered as various sizes of cylinders. In accordance with the resulting numerical data, a 3-D image was reconstructed with software Metasequoia shareware (http://metaseq.net/). (**e**) The data of the reconstructed 3-D images was converted with Autodesk® 3ds Max® 2012 (Autodesk, San Rafael, CA, USA). On the PC, reconstructed 3-D pulmonary images were appeared as left- and right- eye view images and also output to a 3-D monitor

**Fig. 2:**
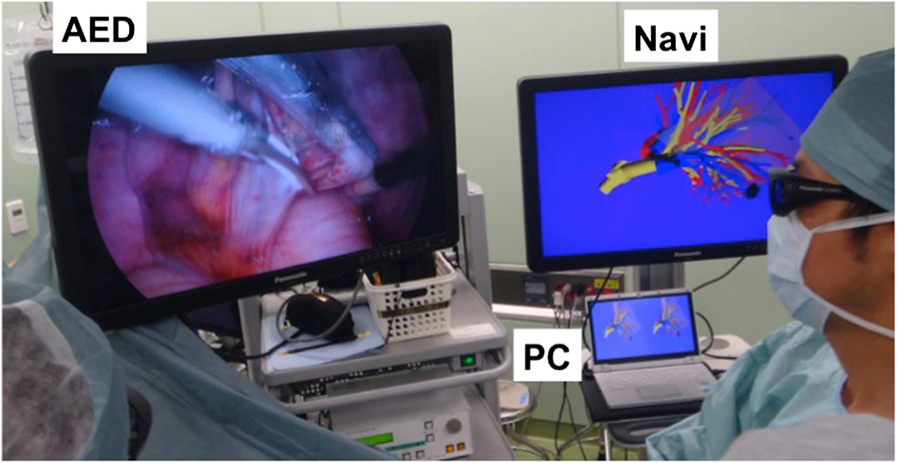
Actual scene in the operating room. Surgeons, nurses, and anesthetists, depending on the case, who wore three-dimensional (3-D) polarized glasses, performed 3-D thoracoscopic sublobar resection, looking at the reconstructed 3-D images on a 3-D monitor for providing binocular stereo-navigation. AED, actual endoscope 3-D display; Navi, binocular stereo-navigation; PC, personal computer

This study was approved by the institutional ethics committee of Tokyo Women’s Medical University (no. 2760). The patients who were 20 years old or older received both oral and written information regarding the procedure. All patients participating in this study were gave informed consent for them to allow the surgeons to use the binocular stereo-navigation system during 3-D TSLRs and the publication of their individual clinical details in publication. From July 2001, our department began to use a virtual 3-D pulmonary model on a personal computer (PC) (CF-AX2, Panasonic, Osaka, Japan) for preoperative simulation before thoracoscopic lung resection and for intraoperative navigation during operation. Furthermore, binocular stereo-navigation during 3-D TSLR has been started from January 2012. All patients were examined by high-resolution computed tomography (HRCT) without contrast agents. The creation of patient-actual virtual 3-D pulmonary model is previously described [12–14]. After 120 of 1-mm thin-sliced HRCT-scan images of tumor and hilum in digital imaging and communications-in-medicine (DICOM) format were uploaded to the PC, homemade software, named “CTTRY” (Tokyo Women’s Medical University, Tokyo, Japan), allowed surgeons to mark pulmonary arteries, veins, bronchi, and tumor on the HRCT images manually and attempted to reconstruct an anatomical model with the help of anatomically correct images (Fig. 1a-c). The locations and thicknesses of the pulmonary vessels and bronchi were rendered various sizes of cylinders. In accordance with the resulting numerical data, a 3-D image was reconstructed by Metasequoia shareware (Metasequoia, http://metaseq.net/) (Fig. 1d). Subsequently, the data of the reconstructed 3-D images by Metasequoia were converted to Autodesk data with Autodesk® 3ds Max® 2012 (Autodesk, San Rafael, CA, USA). Finally, REMO Exporter® (3D Incorporated, Kanagawa, Japan), which can reproduce real-time computer graphics (CG) from Autodesk data, was able to show 3-D CG on a stereoscopic vision display. Reconstructed 3-D pulmonary images appeared as left and right-eye view images on the screen of PC and overlaid 3-D images on a monitor. Surgeons were able to watch the 3-D CG of the reconstructed 3-D pulmonary model on a 3-D liquid crystal (LC) monitor (Panasonic Healthcare, Tokyo) by REMO Viewer® (3D Incorporated) (Fig. 1e). Wearing 3-D polarized glasses, surgeons were able to manipulate the reconstructed 3-D images on a 3-D LC monitor for simulating virtual surgical procedures such as reshaping, cutting, and moving preoperatively. Three-dimensional TSLR was performed by a 3-D digital vision system (Shinko Optical, Tokyo, Japan). Surgeons wearing 3-D polarized glasses performed 3-D TSLR, watching the reconstructed 3-D images on a 3-D LC monitor intraoperatively (Fig. 2). The surgical procedure was performed under general anesthesia with single-lung ventilation, and the patient was allowed to be in the lateral decubitus position. Depending on segments or subsegments that would be removed surgically, an operating surgeon stood to face patient’s anterior chest or back. A 2.5-to-5-cm access incision was placed in the anterior axillary line in the 4th or 5th intercostal space (ICS). An assist port was placed at the midaxillary line of the 7th or 8th ICS, and a camera port was placed along the same ICS in the posterior axillary line. A rigid 30° endoscope was used. The segments were divided by the combined used of an electrocautery, ultrasonically activated device, and a stapler. All patients with primary lung cancer were preoperatively staged as N0 by HRCT of the chest and positron emission tomography.

## Results

The clinical and operative characteristics in this study are showed in Tables 1 and 2. The patients consisted of 5 men and 5 women, ranging in age from 65 to 84 years (mean: 76.3 ± 6.6 years). The clinical diagnose were a primary lung cancer in 6 cases and a solitary metastatic lung tumors in 4 cases. Tumor locations were the right upper lobe in three patients, the right lower lobe in 1, the left upper lobe in 3, and the left lower lobe in 3. The tumor sizes were measured to be less than 10 mm in one patient, 11 to 15 mm in four, 16 to 20 mm in four, and 21 to 25 mm in one. TSLRs were performed with the intraoperative assessment of lymph nodes. Eight cases of single segmentectomy were performed in two cases of the right S1, the left S1 + 2, and the left S10, and in one case of the right S2 and the right S8. One case of bi-segmentectomy was performed in the left S9 + S10. One bi-subsegmentectomy was performed in the left S1 + 2c + S3a. Although intraoperative assessment of lymph nodes, hilar lymphadenectomy with mediastinal lymph node sampling, has been performed in 6 primary lung cancer, four patients who underwent a single segmentectomy with metastatic lung tumors were performed without lymphadenectomy. The operation time ranged from 125 to 333 min (mean: 204.8 ± 60.1 min). The estimated blood loss ranged from 5 to 187 g (mean: 45.5 ± 56.5 g). There were no intraoperative complications and no conversion from TSLR to open thoracotomy and lobectomy. No patient needed a blood transfusion during peri-operative period. Postoperative courses of eight patients were an uneventful, and other two patients had a prolonged lung air leak. The drainage duration ranged from 2 to 13 days (mean: 4.7 ± 3.6 days), and the hospital stay ranged from 8 to 19 days (mean: 12.1 ± 3.6 days). The tumor histology of primary lung cancer showed 5 adenocarcinomas and 1 squamous cell carcinoma. All patients with primary lung cancer were at stage IA. The organs with metastatic pulmonary tumor were kidney, bladder, breast, and rectum. No patients had macroscopically positive surgical margins.

**Table 1:** The clinical characteristics

Number (%)	10 (100)
Age, Mean ± SD (years)	76.3 ± 6.6 (range, 65.0–84.0)
Sex, *n* (%)	
Men	5 (50)
Women	5 (50)
Clinical diagnoses, *n* (%)	
Primary lung cancer	6 (60)
Metastatic lung tumors	4 (40)
Tumor locations, *n* (%)	
Right upper lobe	3 (30)
Right lower lobe	1 (10)
Left upper lobe	3 (30)
Left lower lobe	3 (30)
Tumor sizes, *n* (%)	
≤10 mm	1 (10)
11–15 mm	4 (40)
16–20 mm	4 (40)
21–25 mm	1 (10)

**Table 2:** The operative characteristics

Surgical procedures, *n* (%)	
Singe segmentectomy	8 (80)
Resected sites	
Right S1	2 (20)
Right S2	1 (10)
Right S8	1 (10)
Left S1 + 2	2 (20)
Left S10	2 (20)
Bi-segmentectomy (Left S9 + S10)	1 (10)
Bi-subsegmentectomy (Left S1 + 2c + S3a)	1 (10)
Lymphadenectomy, *n* (%)	
Hillar with mediastinal LN sampling	6 (60)
None	4 (40)
Operation time, mean ± SD (min)	204.8 ± 60.1 (range, 125–333)
Estimated blood loss, mean ± SD (g)	45.5 ± 56.5 (range, 5–187)
Drainage duration, mean ± SD (days)	4.7 ± 3.6 (range, 2–13)

LN lymph node

## Discussion

Conventional open thoracotomy procedure is replaced with thoracoscopic lung resection, which is performed for many pleuro-pulmonary diseases in our department. Only well experienced thoracic surgeons, who understand of variation in the lung anatomy, resect the lung by VATS with the help of a two-dimensional (2-D) LC monitor without problems [13, 14]. Many experienced thoracic surgeons acquire their knowledge of variation in the anatomy through open thoracotomy. On the other hand, inexperienced thoracic surgeons, such as resident physicians and junior surgeons, have to learn surgical anatomy by VATS with a 2-D LC monitor. Therefore, software CTTRY is developed for educating inexperienced thoracic surgeons about the precise lung anatomy [12]. CTTRY allows them to trace and mark the anatomical structure, such as the pulmonary arteries, pulmonary veins, bronchi, and tumor, on the HRCT images by a hand-assisted definition. CTTRY also allows patients’ specific preoperative assessments and virtual planning to be realized. Although a few systems reconstructing 3-D images from CT images are known, those systems are almost expensive, difficult to use, and difficult to be modified by users including medical doctors. As its advantage, CTTRY is inexpensive and uses CT images without contrast agents, and the modification of the software is easy because of homemade software. On the other hand, as a disadvantage, longer reconstruction time, approximately 30 min, is necessary. Because CTTRY is developed for surgical anatomy education, reconstructing time for 3-D image was out of concern at the development time.

Patient’s personal reconstructed 3-D pulmonary image by CTTRY is unexpectedly quite similar to intraoperative thoracoscopic view and can identify the bronchovascular structures accurately [12–15]. Therefore, TSLR can be performed with pulmonary vessels and bronchi oriented away from the tumor.

Anatomic variations of the pulmonary artery and pulmonary vein have a potential risk of uncontrollable bleeding intraoperatively, and especially vascular injury is associated with a limited field of vision during thoracoscopic procedure [12]. Based on these backgrounds, reconstructed 3-D pulmonary images by CTTRY are used for VATS as an intraoperative navigation tool. Preoperative 3-D image provides valuable information to surgeons who want to simulate the planned lung resection [8–14]. Reconstructed 3-D image gives the anatomic relationship among the pulmonary arteries, pulmonary veins, bronchi, and the tumor to surgeons in thoracoscopic surgery. Reconstructed 3-D pulmonary image is useful not only in educating anatomical variations but also in preoperative simulation and intraoperative navigation [16].

Our humans live in 3-D space and want to obtain image information in 3-D format for recognizing image information surely. With remarkable advances in computer science, a 3-D image can be made with CG data from chest CT image data easily. Being expressed by CG technology, virtual 3-D image is displayed on PC screen as a 2-D image. Recently, a 3-D display becomes inexpensive, and patient’s personal 3-D image can be displayed on a large 3-D monitor easily [17]. With CTTRY data, intraoperative binocular stereo-navigation was used for 3-D thoracoscopic lung resection. After being inexpensive, 3-D thoracoscope was introduced in the authors’ department. Three-dimensional thoracoscopic operation with binocular stereo-navigation really improved depth perception and gave an intraoperative view, which was more similar to that of an open thoracotomy than that of a conventional thoracoscopic surgery.

A 3-D printed model for prototyping begins to attract attention recently, but 3-D printers are still expensive, discouraging the popularization of the printer [18]. Although a 3-D printed model gives a real 3-D image, the binocular stereo image also gave an artificial 3-D image, which is sufficient for thoracoscopic lung resection. This study could prospectively show that the binocular stereo-navigation was suitable for performing TSLR with a sufficient margin for small pulmonary tumors.

## Conclusion

This study investigated the efficacy of binocular stereo-navigation during 3-D TSLR. The binocular stereo-navigation was able to identify the bronchovascular structures accurately and was suitable for performing TSLR with a sufficient margin for small pulmonary tumors.

## Abbreviations

CT: Computed tomography
VATS: Video-assisted thoracic surgery
TSLR: Thoracoscopic sublobar resection
3-D: Three-dimensional
PC: Personal computer
HRCT: High-resolution computed tomography
DICOM: Digital imaging and communications in medicine
CG: Computer graphics
LC: Liquid crystal, S, segment
2-D: Two-dimensional
